# Endophytic *Bacillus vallismortis* and *Bacillus tequilensis* bacteria isolated from medicinal plants enhance phosphorus acquisition and fortify *Brassica napus* L. vegetative growth and metabolic content

**DOI:** 10.3389/fpls.2024.1324538

**Published:** 2024-03-22

**Authors:** Aziza Nagah, Mostafa M. El-Sheekh, Omnia M. Arief, Mashael Daghash Alqahtani, Basmah M. Alharbi, Ghada E. Dawwam

**Affiliations:** ^1^ Botany and Microbiology Department, Faculty of Science, Benha University, Benha, Egypt; ^2^ Botany Department, Faculty of Science, Tanta University, Tanta, Egypt; ^3^ Department of Biology, College of Science, Princess Nourah bint Abdulrahman University, Riyadh, Saudi Arabia; ^4^ Biology Department, Faculty of Science, University of Tabuk, Tabuk, Saudi Arabia; ^5^ Biodiversity Genomics Unit, Faculty of Science, University of Tabuk, Tabuk, Saudi Arabia

**Keywords:** biofertilization, canola, phosphate solubilization, *Bacillus vallismortis*, *Bacillus tequilensis*, metabolic content

## Abstract

Phosphorus fertilization imposes critical limitations on crop productivity and soil health. The aim of the present work is to explore the potential of two phosphate solubilizing bacteria (PSB) species in phosphorus supplementation of canola (*Brassica napus* L.). Out of 38 bacterial isolates obtained from nine medicinal plants, two bacterial strains (20P and 28P) were proved as the most potent for the *in-vitro* tricalcium phosphate solubilization test. These isolates verified their activity toward different enzymes as nitrogenase and alkaline phosphatase. Also, 20P and 28P gave a high amount of indole-3-acetic acid, 34.16 μg/ml and 35.20 μg/ml, respectively, and were positive for siderophores production as they detected moderate affinity for iron chelation. Molecular identification confirmed that strain 20P was *Bacillus vallismortis* and strain 28P was *Bacillus tequilensis*. A pot experiment was conducted to study the effect of four different phosphorus concentrations (0%, 50%, 75%, and 100% P) each alone and/or in combination with *B. vallismortis*, *B. tequilensis*, or both bacterial isolates on the vegetative growth and some physiological parameters of canola. The combined treatment of 50% phosphorus + (*B. vallismortis* + *B. tequilensis*) was generally the most effective with respect to shoot height, shoot dry mass, leaf area, photosynthetic pigment fractions, total sugar content, and accumulated NPK content. In contrast, the rhizosphere pH reached the minimum value under the same treatment. These findings highlighted the potential use of PSB (*B. vallismortis* and *B. tequilensis*) along with phosphorus fertilization as a safe sustainable tactic.

## 1 Introduction

In this contemporary epoch, cooking oils suffer an unprecedented leap in prices, which becomes one of the most critical challenges for consumers. Canola (*Brassica napus* L.) or oilseed rape as it is known elsewhere is a promising oilseed crop. Canola comes after soybean and palm oil in relation to oilseed crop production worldwide (Abdel Latef, 2011; Nobile et al., 2019). Above and beyond, canola is categorized as the first in terms to stress tolerance among the field oil crops (Lohani et al., 2020). The economic importance of canola seeds lies in their nutritional content represented by 25% protein and 40%–42% oil including 60% oleic acid and 8.8% linoleic acid (Milazzo et al., 2013; Carré and Pouzet, 2014).

Canola farming in Egypt offers a chance to overcome a number of manufacturing challenges with cooking oils. Furthermore, in order to avert opposition with other crops occupying the old cultivated lands, canola could be successfully grown in newly reclaimed land outside the old Nile Valley zones (ELSabagh et al., 2015). The nutritional status of the soil has a significant impact on the success of canola farming and any crop. Fertile soil is crucial for plants, because it contains vital nutrients as well as a diverse and influential biotic population that keeps the soil from ecological factors (Pereira and Da Gama, 2008). Enhancing the availability of soil nutrients to crop plants such as for oil and grain production is of great significance (Salimpour et al., 2010). Phosphorus (P) is an indispensable nutrient in the soil–plant nutrient cycling (Cheng et al., 2023). This macronutrient plays a pivotal role in plants at both physiological and biochemical levels during critical growth phases (Marschner, 2012; Malhotra et al., 2018). In addition to other metabolic pathways, P plays a role in the metabolism of phospholipids, adenosine triphosphate, and nucleic acids in plant cells (Poirier et al., 2022).

The productivity of several agroecosystems is hampered by soil P shortage (Reijnders, 2014). By means of root transporters, plants take up P from the soil; however, the accessible forms of P are restricted in rhizospheres (Bidondo et al., 2012). Thus, external amendment of chemical P fertilizers could satiate crop P demands during crucial growth phases (Fixen and Johnston, 2012; Cheng et al., 2023). The primary method of phosphorus amendment is the use of mineral fertilizer composed of phosphate rock (Reijnders, 2014). However, in soils that have insufficient P, certain microbes and plants can mobilize unavailable P along with organic waste (Richardson et al., 2011; Robles-Aguilar et al., 2019).

Khan (2020) revealed that there is a hesitation of the unjustified usage of synthetic fertilizers and pesticides in farming without ecological costs. This issue is under discussion due to ecological concerns and consumers’ health fears. The excessive use of chemical fertilizers causes dramatic anxiety including changing soil pH resulting in its acidity, reduction in soil organic matter content, and contributes to the release of greenhouse gases (Kumar et al., 2019). Excessive consumption of P fertilizers leads to low-phosphorus acquisition efficiency. This has severe consequences for the environment and speeds up the exhaustion of this non-renewable P reserve by the end of the century (Fixen and Johnston, 2012; Cheng et al., 2023). The role of the researchers in increasing the life span of world phosphorus reserves lies in increasing the efficiency of P use in agriculture (Syers et al., 2008). This efficiency is around 10%–25% worldwide. In spite, the accessible P concentration in soil is 1 ppm, which is critically low (Khan et al., 2009). Precipitation reactions with the highly reactive Ca^2+^ in typical or calcareous soils cause significant amounts of P applied through fertilization to disperse to immobile pools (Gyaneshwar et al., 2002). Amorphous aluminum (Al), iron (Fe), or manganese (Mn)–bound P are other inorganic P forms (Xie et al., 2011). All of these phosphate-containing rock minerals frequently lack the solubility necessary to provide crops with adequate P requirements, so they must be converted into readily usable P forms (Houben et al., 2019).

From the previously mentioned and in light of the food crisis and climatic changes that strike the world everywhere, numerous researchers nominated green cultivation to encounter P shortage and sustain the dynamic biotic population. This strategy aims to offer more accessible P while restricting the excessive use of the mineral fertilizer (Faucon et al., 2015). The term bio-fertilization involves applying beneficial microorganisms or their metabolites in order to enhance soil biological and chemical properties, maintain soil fertility, and promote plant growth and productivity (Suleiman et al., 2020; Ammar et al., 2022). This application is a reliable alternative sustainable solution to displacing chemical fertilizers without ecological losses (Khan, 2020).

According to Hussain et al. (2016) and NAMLI et al. (2017), phosphate solubilizing bacteria (PSB) are used by the agro-industry in combination with applied phosphates to access more P and boost crop yields. For instance, PSB have the potential to convert insoluble phosphate into a more accessible form to plants through solubilization and mineralization processes (Cheng et al., 2023). For that, PSB became an alternative to chemical phosphatic fertilizer (Khan et al., 2007). Generally, colonization of beneficial microorganisms promotes root system expansion to enhance nutrient acquisition, thus enhancing the general performance of plants in terms of growth and productivity of crops (Kumar and Dara, 2021; Pirttilä et al., 2021). PSB not only carry out a crucial part in plant nutrition in agriculture but also make soluble phosphorus accessible to subsequent plants (Elhaissoufi et al., 2022). Bhattacharyya and Jha (2012) documented that several genera, including *Bacillus* species, contain the most promising PSB. These soil bacteria carry out a major task in the movement of soil P by secreting a number of organic acids, which dissolve P minerals, rendering P more accessible to roots (Sarker et al., 2014). According to Guang-Can et al. (2008), *Bacillus* species have the capacity to mineralize 8–18 g mL^−1^ of organic P and to solubilize 25–42 g mL^−1^ of inorganic P.

This investigation is constructed to evaluate the inspiration of seed presoaking and irrigation using PSB to facilitate the mobility of P for canola crops. To achieve this goal, we used liquid cultures of the two bacilli strains: *Bacillus vallismortis* strai*n* 20P and *Bacillus tequilensis* strain 28P. Hence, canola species may increase both their own growth, as well as the subsequent crops, because of the increased P mobilization in the soil.

## 2 Materials and methods

### 2.1 Bacteriological assessments

#### 2.1.1 Sample collection and bacterial isolation

Nine medicinal plants (**Supplementary Table S1**) were gathered from different farms at Qalyubiya Governorate (30°18'0”N/31°15'0”E), Egypt. The healthy parts were chosen for the isolation of endophytic bacteria. The samples were separated into roots, stems, and leaves before being thoroughly rinsed with tap water to remove any clingy soil. The plant components were thoroughly cleaned and cut into 0.5–1 cm segments and subsequently sterilized with 70% ethanol and 2% sodium hypochlorite for 30 s and 5 min, respectively. The samples were then cleaned twice in a laminar flow hood with sterile distilled water (Elbeltagy et al., 2000). The sterilized components were ground in 0.8% saline solution and quartz sand, and they were then diluted decimally in 0.8% saline solution. Sterility tests were carried out to confirm that the isolated strains originated from endophytes (the inside of the plant). The final dilutions (10^−4^ and 10^−5^) were applied to specific cultural medium, Pikovskaya’s (PVK) agar medium (Pikovskaya, 1948). Bacterial plates were parafilm sealed and cultured at 37°C for 48h. Pure cultures were kept for future research after the incubation time on slants at 4°C.

#### 2.1.2 Quantitative assessment of phosphate solubilization

For quantitative analysis of phosphate solubilization, bacterial isolates were transferred to the National Botanical Research Institute’s phosphate (NBRIP) broth medium (Nautiyal, 1999) and then incubated for 7 days at 28°C. The cultures were subjected to centrifugation at 3885 RCF for 20 min, and the phosphorous concentration in the culture supernatant was detected by molybdenum blue colorimetric method (Olsen and Sommers, 1982). Using Spekol Spectrophotometer VEB Carl Zeiss set to 600 nm, the color was evaluated (Naik et al., 2009). For quantitative analysis, a standard curve was constructed using potassium dihydrogen phosphate (KH_2_PO_4_) solution. At the completion of the experiment, the ultimate pH of the media was determined by digital pH meter.

#### 2.1.3 Screening for other plant growth-promoting traits

Different plant growth-promoting (PGP) traits as alkaline phosphatases, indole-3-acetic acid (IAA), siderophores production, HCN production, and nitrogenase activity were also detected. These traits were assessed for the most potent isolates (20P and 28P) that showed high efficiency for quantitative phosphate solubilization. According to Tabatabai and Bremner (1969) technique, alkaline phosphatases were measured using p-nitrophenyl phosphate, a colorless substrate that produces the yellow-colored end product p-nitrophenol. The absorbance at 420 nm was employed to quantify the concentration of p-nitrophenol in triplicates using a spectrophotometer. Values were estimated using a standard curve created using p-nitrophenol serially diluted solutions as the standard. The quantity of enzyme required to release 1 mol of p-nitrophenol/ml/min from di-Na p-nitrophenyl phosphate (tetrahydrate) under the test conditions was designated as one unit (U) of phosphatase activity.

Detection and quantification of IAA was implemented along with the technique outlined by Gordon and Weber (1951). The emergence of a pink–red color was used to detect IAA generation, and the absorbance at 530 nm was quantified using a spectrophotometer. In order to create a standard curve for quantitative analysis, color was produced in a standard solution of pure IAA (Sarwart et al., 1992).

Siderophores production was estimated by adding 8-hydroxyquinoline (50 mg/L) as iron chelators to tryptic soy agar (TSA) medium (Alexander and Zuberer, 1991). Freshly produced cultures were used to inoculate plates of TSA media, which were then incubated for 3–7 days at 28°C. Ability of isolates to grow on this medium was considered as a positive result for siderophores production.

In addition, the ability of isolates toward nitrogen fixation was evaluated by acetylene reduction assay (ARA) according to Hardy et al. (1973). Each bacterial isolate was inoculated in a 25-mL flask containing 10 mL of JNFb medium and incubated for 3 days at 30 ± 2°C. Inside air from the tubes is replaced with 5 mL of acetylene through a syringe and the tubes were incubated for 24h at 30 ± 2°C. The nitrogenase activity was assessed by ARA of mid-log phase broth cultures after 24h.

For assessing the antagonistic activity between bacterial isolates, a single bacterial isolate was inoculated in lines over the surface of the nutrient agar medium. Along lines perpendicular to the first inoculations, the second isolate was inserted. After an incubation period of 24h at 37°C, the growth inhibition zones were recorded (Seyfferth et al., 2021).

#### 2.1.4 Molecular identification

##### 2.1.4.1 DNA extraction and analysis of molecular phylogeny using the 16S rRNA gene sequence

According to the instructions provided by the GspinTM Total Extraction kits, the genomic DNA for 20P and 28P was obtained. Cells cultured on LB broth were used for the bacterial DNA extraction. Thermocycler was then used to amplify the new isolates’ 16S rRNA genes (Biometra thermocycler, Germany) using the universal primers 27F 5’ (AGA GTT TGA TCM TGG CTC AG) 3’ and 1492R 5’ (TAC GGY TAC CTT GTT ACG ACT T) 3’ as designated by Pandey et al., (2002). The 20 μl of polymerase chain reaction (PCR) contained 0.2 μl of 2.5 U/μl Taq DNA polymerase, 2 μl of 10× buffer, 1.6 μl of 2.5 mM dNTPs, 1 μl Primer F, R (10 pmole/μl), 1 μl of template (20 ng/μl), and up to 20 μl of distilled water [High-performance liquid chromatography (HPLC) grade]. The PCR amplification condition includes an initial denaturation at 95°C for 5 min, subsequently 30 cycles (95°C denaturation for 30 s, 55°C annealing for 2 min, 68°C extension for 1.5 min), and final extension at 68°C for 10 min. Purification of PCR products was performed using Montage PCR clean-up kit (Millipore). Purified PCR products were treated with BigDye Terminator Cycle Sequencing Kit v.3.1 (Applied Biosystems, USA), and the sequencing products were run on an Applied Biosystems Model 3730XL automated DNA sequencing system (Applied Biosystems, USA) at the Macrogen, Inc., Seoul, South Korea. The Sequence Similarity Search was disclosed for the 16S rDNA sequence using an online search tool (http://www.ncbi.nlm.nih.gov/blast/). The unknown organisms (20P and 28P) were identified using the maximum aligned sequence through BLAST search and deposited in GenBank under accession numbers (OM978276.1 and OM978277.1), respectively.

### 2.2 Plant experiment

#### 2.2.1 Experiment design and growth conditions

A pot experiment was conducted on 26 September 2021 at the Benha University off-campus botanical garden, under natural conditions for canola growth. Canola seeds were surface sterilized with 0.01% mercuric chloride for 3 min and then thoroughly washed with distilled water. The sterilized seeds were classified into 16 groups as illustrated in **Supplementary Figure 1**. The experiment was set up under completely randomized block design with 160 bags. Each bag filled with 2 Kg washed and sterilized river clay: sand (2:1 V/V) soil. Plants received the recommended doses of phosphorus and potassium fertilizers by mixing with the soil three days before sowing. Superphosphate and potassium fertilizer (15.5% P_2_O_5_ and 48% K_2_SO_4_, respectively) were used. The optimum phosphorus concentrations for canola germination were determined by a preliminary germination experiment as 100%, 75%, and 50% P. These concentrations were achieved by the following recipes: 100% P (0.39 g superphosphate/2 Kg soil mixture), 75% P (0.225 g superphosphate/2 Kg soil mixture), and 50% P (0.195 g superphosphate/2 Kg soil mixture). In addition, urea fertilizer)46% N_2_) was added 21 days after sowing (DAS) to the bags. The bags were maintained at the campus under normal day/night period and irrigated with tap water twice weekly or when needed. Canola seeds were applied to the selected endophytic bacterial inoculum (10^6^ CFU/ml) carboxymethyl cellulose (CMC) solution (5%) in the range of 500 g seeds/125 ml of inoculum mixed with 25 ml of CMC. Regarding co-inoculation application, both bacterial strains were counted separately and then added to the soil. Uniform seeds were left to dryness before planting. Control treatment seeds were retained. After seedling growth, the plants were thinned to four plants per pot, and then each pot was inoculated with 10 ml of bacterial inoculum (10^6^ CFU/ml) of endophytic bacterial strains (*B. vallismortis* and *B. tequilensis*) separately or in combination. Samples of canola plants representative for each treatment were collected 45 days old after sowing (45 DAS), when apparent variations in plant size could be seen between the various treatments. It should be mentioned that, for determination of the different growth parameters, five replicates were used, whereas triplicate samples were analyzed for the chemical analyses.

#### 2.2.2 Evaluation of growth biomarkers

Representative plants from each treatment were taken out of their pots and dipped in a beaker of water to wash away any soil that had stuck to them. The plants were then wiped with tissue paper. A meter scale was used to measure the heights of the shoots. After that, the samples were weighed to determine their fresh masses, and they were then put in an oven that was set to 70°C. After cooling at room temperature, the samples were reweighed many times until we obtained their constant dry masses by the end of 72h. Root mass ratio was calculated as the ratio of root DM to total plant DM. Using a scanner and the ImageJ program, the depth of the roots and the total area of the plant’s leaves were also measured.

#### 2.2.3 Metabolic contents

##### 2.2.3.1 Extraction and estimation of photosynthetic pigments

According to the recommendations of Arnon (1949) and Horvath et al. (1972) as well as the modifications of Kissimon (1999), the quantitative amounts of chlorophylls a and b and carotenoids were calculated. Estimation took place using a spectrophotometer. The extracts were measured in comparison to a blank of pure 80% aqueous acetone at three different wavelengths: 480 nm, 644 nm, and 663 nm. The concentration of each photosynthetic pigment was assessed as μg ml^−1^ according to the subsequent equations, taking into account the dilutions made:


Chlorophyll a=10.3 E663−0.918 E644=µg/ml

Chlorophyll b=19.7 E644−3.87 E663=µg/ml

Carotenoids=5.02 E480=µg/ml

Then, the pigment fractions were expressed as mg g^−1^ dry weight of leaves.

##### 2.2.3.2 Extraction and estimation of carbohydrate fractions

According to Prud’homme et al. (1992), known dried weights were digested. After that, soluble sugars were determined using the anthrone–sulfuric acid procedure as designated by Whistler and Wolfrom (1963). A blue–green color was formed, and it was determined spectrophotometrically at a wavelength of 620 nm in comparison to a blank made up only of water and anthrone reagent. For the purpose of measuring polysaccharides, the dry residue left over after the extraction of soluble sugars was used. The anthrone–sulfuric acid reagent technique was employed for polysaccharides estimation as in the case of soluble sugars. Triplicate samples were analyzed for carbohydrate analyses. Total carbohydrates were mathematically determined as the total of the sample’s soluble sugar and polysaccharide concentrations. The calculations for all sugar fractions were calculated from a calibration curve created using only pure glucose, and the data were expressed as mg glucose g^−1^ dry weight. Triplicate samples were analyzed for the chemical analyses.

##### 2.2.3.3 Extraction and estimation of the macronutrients NPK

By using the traditional semi-micro-modification of the Kjeldahl method (Chibnall et al., 1943), total nitrogen was digested. Following digestion, the Pregi (1945) method was employed to calculate the total nitrogen. The outcomes of the titration were expressed as mg nitrogen using


$$1\text{ ml of }0.0143\text{N }{\text{H}}_{2}{\text{SO}}_{4}=0.28\text{ mg nitrogen}$$

The concentrations of total nitrogen were expressed as mg nitrogen 100 g^−1^ dry weight. Finally, the quantitative amounts of accumulated N were expressed by multiplying the nitrogen concentration (g) by DM (g) (Zhao et al., 2010).

A wet ashing procedure was employed to extract both potassium and phosphorus. The dried tissues were digested using the technique described in Chapman and Pratt (1962).

Utilizing the flame emission method, potassium was calculated (Ranganna, 1977). Whereas, phosphorus was measured with inductively coupled plasma optical emission spectrometry (Soltanpour, 1985). The quantitative amounts of accumulated PK were expressed as the case of accumulated N.

### 2.3 Statistical analysis

Data for quantitative phosphate solubilization (measurements were performed in triplicate) were analyzed on the premise Duncan’s multiple range test at a probability level (*P*) ≤ 0.05 (IBM SPSS Statistics software version 21). Generalized least square models were used (Zuur et al., 2009) to test the response of canola using two bacteria (*Bacillus vallismortis* and *Bacillus tequilensis*) at four P levels and the interaction between these two factors. Normality of data was checked, and transformations of log_10_ were undertaken if required. If this interaction was significant (*P*< 0.05), then post-hoc *Tukey* tests were also performed (RStudio 2023.03.0). Pearson Correlation analysis was performed to determine the relationship between pH of culture media and the amount of solubilized phosphate (RStudio 2023.03.0). A principal component analysis (PCA) was performed on the logarithmic transformed data using factor extraction to rank the different variables affected by the different treatments. The eigenvalue remained greater than 1 after varimax rotation. Correlation matrix figure was fitted by the GraphPad Prism software 9.0 (GraphPad, USA).

## 3 Results

### 3.1 Bacterial isolation

Thirty-eight endophytic bacteria were obtained from nine medicinal plants on PVK media. The number of bacteria recovered from each plant is shown in the **Supplementary Table S1**. Stevia (*Stevia rebaudiana*) roots had the highest number of bacteria, while the least number of bacterial isolates was obtained from the leaves of Mint, Rosemary, Kalanchoe, Lemon, Nerium, and Roselle plants. We obtained 28 and 10 bacterial isolates from roots and leaves, respectively.

### 3.2 Quantitative phosphate solubilization

Endophytic bacterial isolates showed phosphate solubilization values that ranged between 2.09 and 28.14 µg/ml. Isolates code 28P and 20P recorded the highest phosphate solubilization value (28.42 ± 0.32 µg/ml and 20.43 ± 0.09 µg/ml), respectively. In addition, isolates code 7P and 31P gave the least values (2.29 ± 0.14 µg/ml and 2.35^s^ ± 0.10 µg/ml), respectively. The pH of the culture media was found to be dropping. The culture medium’s pH was initially set at 7 and, as shown in **Table 1**, after the incubation time, the pH was reduced to a range of 4.32–4.41 for the media amended with isolates code 20P and 28P, respectively. A strong negative correlation was detected between culture pH and the amount of solubilized phosphate (−0.702, *P<* 0.0001).

**Table 1:** Screening of the 38 endophytic bacterial isolates for phosphate solubilization.

Isolate code	Phosphate concentration (µg/ml)	Final PH
**1P**	2.66^s^ ± 0.05	6.25
**2P**	9.44^k^ ± 0.05	5.09
**3P**	18.26^c^ ± 0.05	5.2
**4P**	18.24^c^ ± 0.08	5.03
**5P**	3.20^r^ ± 0.04	5.7
**6P**	12.79^g^ ± 0.11	4.63
**7P**	2.29^t^ ± 0.14	5.55
**8P**	3.14^r^ ± 0.08	5.29
**9P**	8.37^l^ ± 0.13	4.87
**10P**	7.68^m^ ± 0.29	5.7
**11P**	8.61^l^ ± 0.19	4.79
**12P**	9.35^k^ ± 0.07	5.4
**13P**	11.72^h^ ± 0.15	4.55
**14P**	10.41^i^ ± 0.32	4.74
**15P**	10.17^i^ ± 0.66	4.46
**16P**	13.62^f^ ± 0.19	4.73
**17P**	4.18^p^ ± 0.03	5.02
**18P**	16.35^d^ ± 0.18	4.67
**19P**	3.24^r^ ± 0.09	5.19
**20P**	20.43^b^ ± 0.09	4.41
**21P**	14.19^e^ ± 0.04	4.74
**22P**	6.72° ± 0.07	4.91
**23P**	10.37^i^ ± 0.09	4.68
**24P**	7.16^n^ ± 0.06	5.18
**25P**	10.44^i^ ± 0.18	5.25
**26P**	3.62^q^ ± 0.34	6.17
**27P**	9.77^j^ ± 0.09	5.51
**28P**	28.42^a^ ± 0.32	4.32
**29P**	8.44^l^ ± 0.06	5.55
**30P**	2.49^st^ ± 0.12	5.71
**31P**	2.35^st^ ± 0.10	5.29
**32P**	3.67^q^ ± 0.11	5.67
**33P**	16.35^d^ ± 0.07	4.55
**34P**	3.62^q^ ± 0.26	5.76
**35P**	7.23^n^ ± 0.11	5.29
**36P**	3.19^r^ ± 0.04	5.44
**37P**	3.16^r^ ± 0.17	5.43
**38P**	3.20^r^ ± 0.06	5.41

*Results were expressed as mean ± standard deviation. No statistically significant difference between values in the same column marked by the same superscript lowercase letter (p ≤ 0.05).

The maximum alkaline phosphatase activity was measured at 1.7 ± 0.08 μmol and 2.4 ± 0.16 μmol p-NP mL^−1^ h^−1^ in isolates code 20P and 28P, respectively. For estimating nitrogenase activity, 20P and 28P isolates gave (9.6 ± 0.47 and 32.1 ± 0.60 n moles C_2_H_4_/ml/h) of ARA, respectively. In addition to phosphate solubilization, isolates 20P and 28P also produced a sizable quantity of IAA and siderophore. Endophytic bacterial isolates (20P and 28P) displayed an intense pink color and gave a high amount of IAA (34.16 ± 0.07 μg/ml and 35.20 ± 0.08 μg/ml), respectively. Furthermore, these isolates were positive for siderophores production and detected moderate affinity for iron chelation. The filter paper’s transition from yellow to brown serves as a marker for the presence of HCN. As evidenced by the lack of any color change in the filter paper impregnated with picric acid, 20P and 28P did not exhibit HCN generation.

### 3.3 Molecular identification

**Figure 1:**
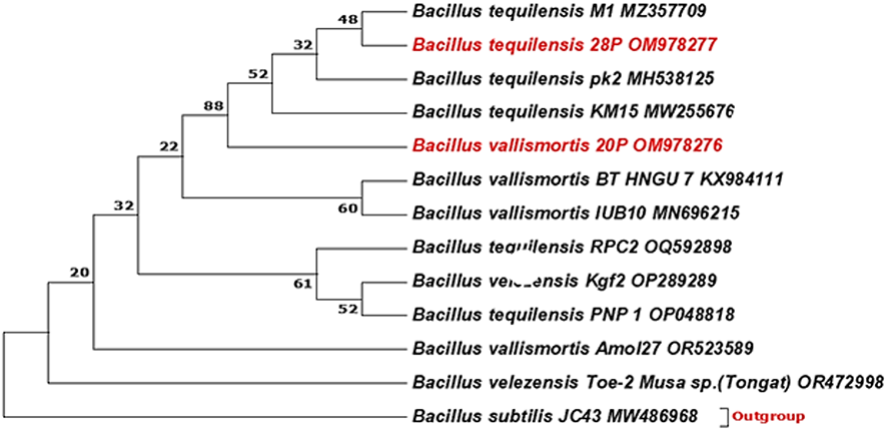
Phylogenetic tree was constructed using the neighbor-joining method. The bootstrap consensus tree inferred from 1,000 replicates is taken to represent the evolutionary history of the taxa analyzed. The percentage of replicate trees in which the associated taxa clustered together in the bootstrap test (1,000 replicates) is shown next to the branches. There was a total of 698 positions in the final dataset. Evolutionary analyses were conducted in MEGA7.

A phylogenetic tree between the designated strains and other strains was exhibited by **Figure 1**. The most effective isolates were identified by molecular identification using 16S rRNA as *Bacillus vallismortis* strain 20P and *Bacillus tequilensis* strain 28P with 100% similarity and recorded in GenBank under accession numbers (OM978276.1 and OM978277.1), respectively.

About the antagonistic activity between the selected bacterial isolates, no antagonism was detected.

### 3.4 Effect of the different phosphorus concentrations and/or the phosphate solubilizing bacteria application on certain morphological parameters, rhizosphere pH, and physiological parameters of canola plant

As general speech, both the single and/or combined phosphorus and bacterial treatments significantly increased all the estimated vegetative and physiological parameters over the control plants. This finding is clearly illustrated by the data tabulated in **Table 2**.

**Table 2:** The effects of phosphorus (P) supplies (0%, 50%, 75%, and 100%) on vegetative growth and physiological characteristics of canola (*Brassica napus* L.) in the absence and/or presence of phosphate solubilizing bacteria PSB; *B. vallismortis*, *B. tequilensis* or both bacterial isolates.

Characteristic	Significance and F value						Marginal means for bacteria			
	P level		Bacteria		P level *Bacteria		B0	*B. vallismortis*	*B. tequilensis*	*B. vallismortis* + *B. tequilensis*
	*P*	F	*P*	F	*P*	F				
**Shoot height (cm)**	ns	1.085	**	5.862	ns	0.541	27.25	28.52	28.6	31.65
**Shoot FM (g)**	ns	1.9380	***	6.9603	ns	0.6943	13.90	14.75	14.55	19.6
**Shoot DM (g)**	ns	2.3636	***	7.2203	ns	0.8136	1.12	1.24	1.24	1.69
**Leaf area (mm^2^)**	ns	1.8554	***	28.8209	*	2.0457				
**Root height (cm)**	*	2.929	***	7.5204	*	2.1087				
**Root DM (g)**	***	24.3937	***	16.2729	***	22.9774				
**Root FM (g)**	ns	4.38264	ns	4.24114	ns	1.78106				
**Root mass ratio (g)**	***	16.8236	***	6.5457	***	16.1989				
**Total DM (g)**	**	4.7021	***	8.166	ns	1.235	1.36	1.52	1.38	1.99
**pH**	***	3.600	***	21.103	***	3.511				
**Chlorophyll a**	*	3.849	***	4.867	**	3.849				
**Chlorophyll b**	*	2.266	ns	0.707	*	3.870				
**Carotenoids**	**	15.398	**	12.260	ns	0.814				
**Total pigments**	*	2.885	ns	1.403	ns	0.334				
**Soluble sugars**	***	16.453	***	26.589	***	1.405				
**Polysaccharides**	***	19.282	***	38.109	***	2.306				
**Total sugars**	***	21.891	***	42.193	***	2.453				
**Accumulated N**	***	0.751	**	5.749	ns	0.799				
**Accumulated P**	***	2.454	***	13.848	***	1.003				
**Accumulated K**	ns	1.014	**	6.158	ns	0.340				

*P< 0.05, ** P< 0.01, *** P< 0.001. DM, dry mass; FM: fresh mass; ns, nonsignificant difference. If there was no significant interaction of P level × bacteria, while bacteria or P level had a significant effect (p ≤ 0.05), then the marginal means for bacteria are presented here. ***p ≤ 0.05.

### 3.5 Ranks of different variables as affected by the different treatments

**Figure 2:**
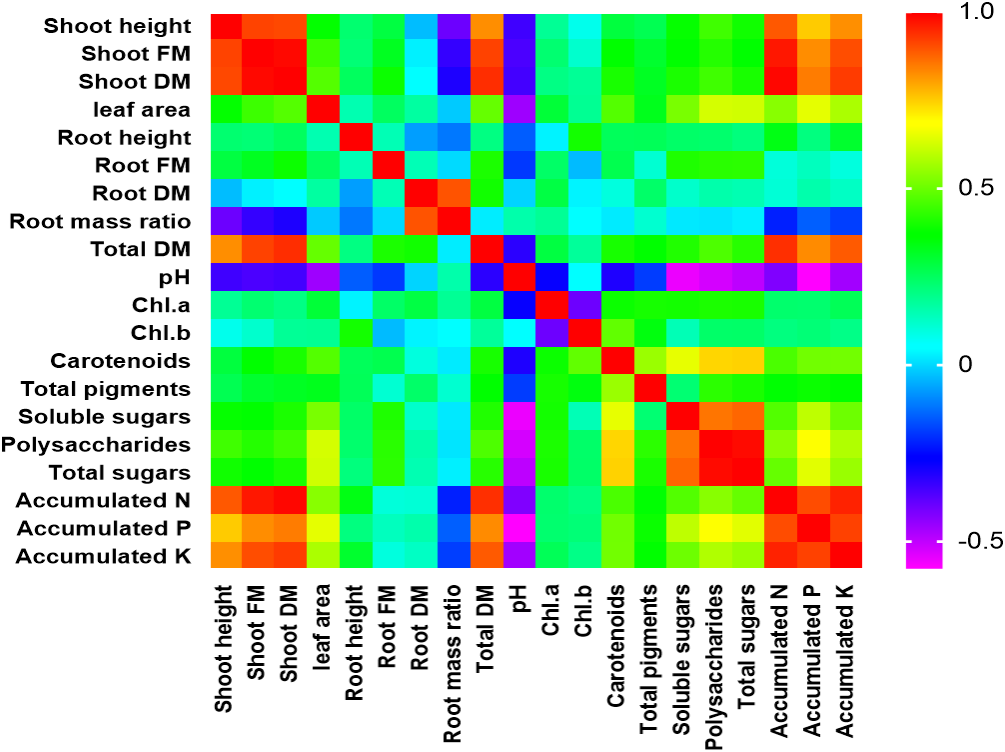
Person correlation matrix of different studied morphological and physiological variables affected by the different concentrations of phosphors (0%, 50%, 75%, and 100% P) loaded by different bacterial isolates (B0, *B. vallismortis*, *B. tequilensis* and *B. vallismortis+ B. tequilensis*).

In this study, PCA and computed eigenvalues had a substantial role in identifying and understanding the associations of different variables affected by the different concentrations of phosphorous combined by different bacterial isolates. Furthermore, the Varimax method was carried out to perform the rotation of the PCA, loadings greater than 0.60 are statistically significant and are marked in bold in **Table 3** and **Figure 2**. The factor analysis included four factors that described 76.95% of total data variability. The first dominant factor accounted for 45.53% of the total variance with an eigenvalue of 9.11. This factor indicated that the shoot height, shoot FM, shoot DM, leaf area, total DM, pH, carotenoids, total pigments, soluble sugars, polysaccharides, total sugars, accumulated N, accumulated P, accumulated K were the major 14 variables affected by the different concentrations of phosphorous combined by different bacterial isolates. The second factor (13.93% of the total variance with eigenvalue = 2.79) revealed that both of root FM and root mass ratio were the second two variables affected by aforementioned different treatments. Just root DM was the third variable affected by the treatment (9.75% of the total variance with eigenvalue = 1.95). Meanwhile, root height, Chl.a, and Chl.b were the latest three variable affected by the treatments (8.27% of the total variance with eigenvalue = 1.65).

**Table 3:** Varimax rotated principle component analysis (PCA) for different morphological and physiological variables affected by the different concentrations of phosphors (0%, 50%, 75%, and 100% P) loaded by different bacterial isolates (B0, *B. vallismortis*, *B. tequilensis* and *B. vallismortis+ B. tequilensis*).

Variables	PC1	PC2	PC3	PC4
**Shoot height**	**0.809**	−0.408	0.153	−0.080
**Shoot FM**	**0.853**	−0.460	0.140	−0.054
**Shoot DM**	**0.878**	−0.429	0.150	0.007
**Leaf area**	**0.670**	0.188	−0.156	−0.083
**Root height**	0.378	−0.046	−0.087	**0.673**
**Root FM**	0.283	**0.679**	−0.044	−0.074
**Root DM**	0.188	0.471	**0.828**	0.035
**Root mass ratio**	−0.101	**0.626**	0.725	0.124
**Total DM**	**0.867**	−0.226	0.427	0.018
**pH**	**−0.601**	−0.125	0.278	0.338
**Chl.a**	0.389	0.418	0.160	**−0.602**
**Chl.b**	0.260	0.056	−0.129	**0.906**
**Carotenoids**	**0.671**	0.409	−0.232	0.289
**Total pigments**	**0.694**	0.268	0.110	0.315
**Soluble sugars**	**0.708**	0.440	−0.296	−0.130
**Polysaccharides**	**0.789**	0.460	−0.273	−0.030
**Total sugars**	**0.757**	0.498	−0.297	−0.028
**Accumulated N**	**0.924**	−0.345	0.120	0.000
**Accumulated P**	**0.924**	−0.134	0.014	−0.057
**Accumulated K**	**0.923**	−0.259	0.085	−0.020
**Eigenvalue**	9.11	2.79	1.95	1.65
**Proportion of variance**	45.53%	13.93%	9.75%	8.27%
**Cumulative proportion of variance**	45.53%	59.46%	69.21%	77.49%

*Bold loadings are statistically significant.

**Figure 3:**
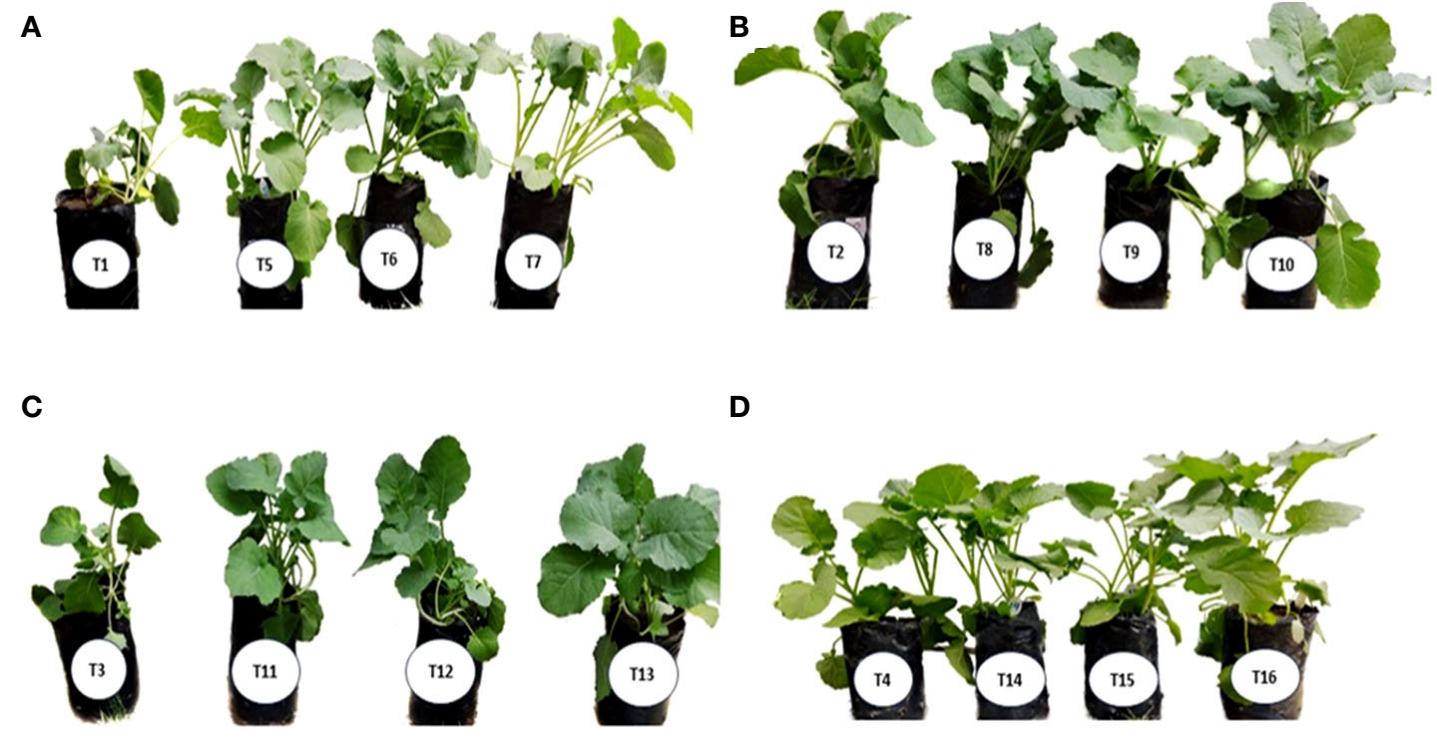
The effect of different concentrations of phosphorus (0%, 50%, 75%, and 100% P) alone and/or in combination with *B. vallismortis*, *B. tequilensis* or both bacterial isolates. on the vegetative growth of canola (*Brassica napus L*.) plant: **(A)** 0% P, (T1): B0, (T5): *B. vallismortis*, (T6): *B. tequilensis* and (T7): *B. vallismortis* + *B. tequilensis*; **(B)** 50% P, (T2): B0, (T8): *B. vallismortis*, (T9): *B. tequilensis* and (T10): *B. vallismortis* + *B. tequilensis*; **(C)** 75% P, (T3): B0, (T11): *B. vallismortis*, (T12): *B. tequilensis* and (T13): *B. vallismortis* + *B. tequilensis*; and **(D) **100% P, (T4): B0, (T14): *B. vallismortis*, (T15): *B. tequilensis*, and (T16): *B. vallismortis* + *B. tequilen*.

**Figure 3** shows the vegetative growth of canola (*Brassica napus L*.) plant under the different concentrations of phosphorus (0%, 50%, 75%, and 100%) alone and/or in combination with *B. vallismortis*, *B. tequilensis* or both bacterial isolates. Shoot height and shoot dry mass (*P ≤* 0.01) differed significantly among the interaction bacteria × *P* levels (*P* ≤ 0.001). The higher values were recorded for the combined treatment *B. vallismortis* + *B. tequilensis* under 50% P. These enhancements were evaluated by 31.37% and 140. 9% for shoot height and dry mass, respectively, over the control treatment; 0% P and 0 bacteria (**Supplementary Table S2**). In the same trend, the leaf area fluctuated significantly between P levels under the combined treatment *B. vallismortis + B. tequilensis*; that of 50% P was greater by 200.8% than the control treatment. The P level treatments brought about a slight modification in the total leaf area of all the other bacterial treatments (B0, *B. vallismortis*, and *B. tequilensis*), but there was no significant difference between P level treatments (**Supplementary Table S2**).

In contrast to the shoot system morphological parameters, root height, root dry mass, and root mass ratio exhibited the highest significant values under both 75 and 100% P + *B. tequilensis*, 50% P + *B. vallismortis*, and 75% +*B. vallismortis*, respectively (**Supplementary Table S2**). The use of the two bacterial strains, *B. vallismortis and B. tequilensis* had significantly improved the total dry mass under all P levels, especially 50% level. Under 50% P combined with (*B*. *vallismortis + B. tequilensis*), an augmented total dry mass estimated by 178.3% over the control plants was achieved (**Supplementary Table S2**).

**Figure 4:**
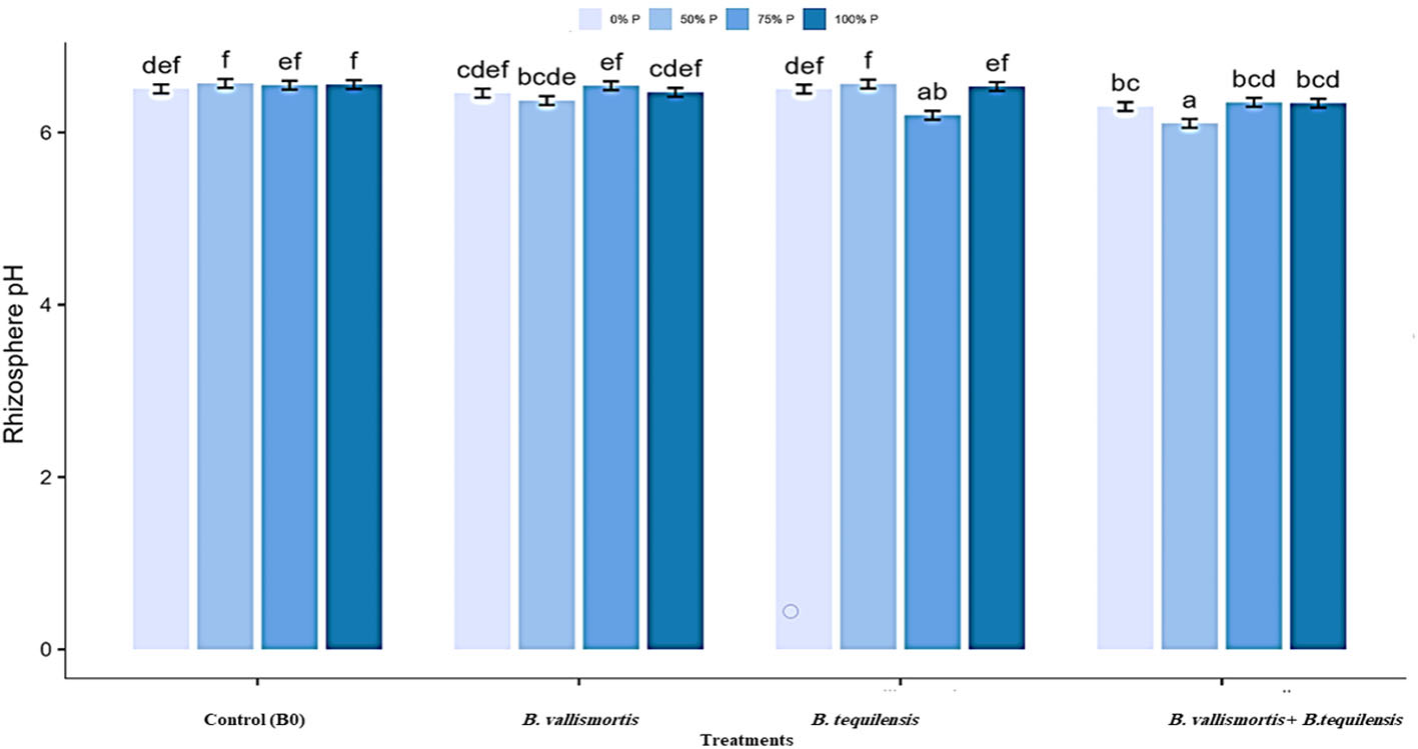
Rhizosphere pH of Canola inoculated with PSB (*B. vallismortis* and/or *B. tequilensis*) shown at (x-axis) under four levels of P (0%, 50%, 75%, and 100% P) shown at *y*-axis. Bars are the means, and error bars are the standard errors of the mean, derived from generalized least square models. Different letters among treatments indicate significant difference within each panel (post-hoc Tukey test, *P* ≤ 0.05). The mean of three replicates (*n* = 3), See **Table 2** for details of the statistical analysis.

The pH of rhizosheath soil varied significantly (**Table 2** among all treatments and interaction between P level and bacterial treatments. The minimum soil pH of 6.11 was acquired for 50% P + (*B. vallismortis* + *B. tequilensis*) treatment. Whereas, the B0, *B. vallismortis*, and *B. tequilensis* treatments showed a bulk soil pH of 6.46, 6.50, and 6.30 under the same P level. The least pH value obtained at 50% P interacted with *B. vallismortis + B. tequilensis* was reduced by 6.1% less than the control in the acidic side (**Figure 4**).

**Figure 5:**
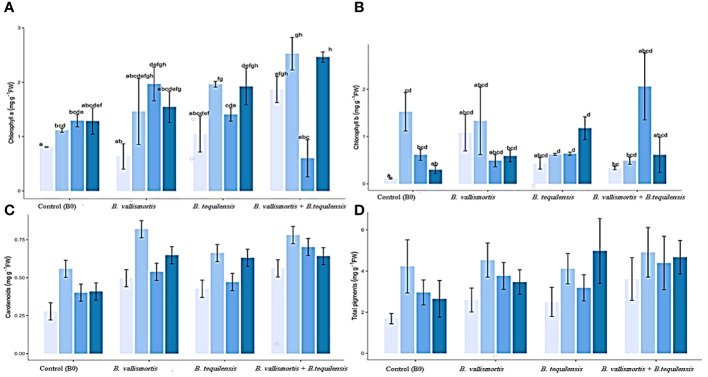
Effect of inoculation with PSB under four levels of P (0%, 50%, 75%, and 100% P) on photosynthetic pigments (mg/g leaves F.wt) of Canola inoculated with PGR shown at (x-axis) under four levels of P Shown at (y-axis). Bars are the means, and error bars are the standard errors of the mean, derived from generalized least square models **(A)** chlorophyll a; **(B)** chlorophyll b; **(C)** carotenoids; and **(D)** total pigments. The mean of three replicates (*n* = 3). See **Table 2** for details of the statistical analysis.

With respect to photosynthetically active pigments content, **Table 2** exposed that all the PSB inoculation treatments could significantly improve the pigment content. The co-inoculation of the two potent PSB *strains B. vallismortis* and *B. tequilensis* and their synergistic interaction caused a positive effect on the pigments’ synthesis. In this connection, chlorophyll a and chlorophyll b, under the bacterial treatment (*B. vallismortis* + *B. tequilensis*), exhibited the greatest amount alongside with 50% P and 75% P in the same order (**Figures 5A, B**). This treatment brought about a significant increase (**Table 2**) reaching more than two and 10-folds above the control for Chl.a and Chl.b, respectively. The proportion of the carotenoids differed significantly among P level and bacterial treatments, with values ranging from 0.56 to 0.66 mg/g F.wt of canola leaves under 50% P for both B0 and *B. vallismortis* + *B. tequilensis* passing by B*. tequilensis.* Furthermore, carotenoids augmented dramatically under the same P level combined with the single treatment of *B. vallismortis* treatment to record 0.82 mg/g D. wt of canola leaves (**Table 2** and **Figure 5C**). The fluctuated responses of the pigment fractions reflected on the total pigments content. The later exhibited the maximum level under 100% p level shared with the inoculation by *B. tequilensis* to achieve 196.4% rise over the control plants. (**Table 2** and **Figure 5D**).

**Figure 6:**
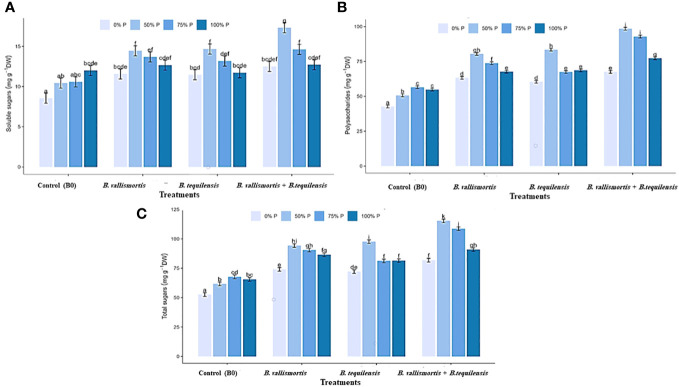
Effect of inoculation with PSB under four levels of P (0%, 50%, 75%, and 100% P) on sugar content of Canola Sp., **(A)** soluble sugars, **(B)** polysaccharides, and **(C)** total sugars. Values are expressed as mg glucose/g DW bars are the means, and error bars are the standard error of the means, derived from generalized least square models the mean is of three replicates (*n* = 3). See **Table 2** for details of the statistical analysis.

**Figure 7:**
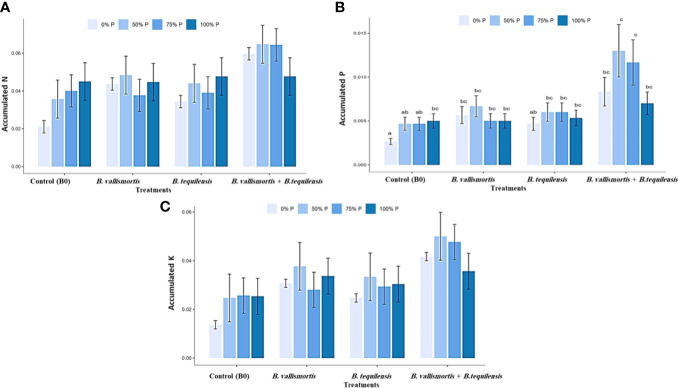
Effect of inoculation with PSB under four levels of P (0%, 50%, 75%, and 100% P) on the accumulated NPK content in plant mature leaf of Canola Sp. **(A)** accumulated N (g) **(B)** accumulated P (g) and **(C)** accumulated K(g). Bars are the means, and error bars are the standard error of the means, derived from generalized least square models, the mean is of three replicates (*n* = 5). See **Table 2** for details of the statistical analysis.

Similar to leaf photosynthetic pigments, soluble sugars differed significantly among P level, bacteria, and interaction between P level × bacteria treatments (**Table 2**). Under 50% P level treatments, soluble sugars varied greatly and an increase was obtained by 68.95% for *B. vallismortis* and 71.5 for *B. tequilens*is and by 102.6% for *B. vallismortis + B. tequilensis* treatments (**Figure 6A**). Polysaccharides also differed significantly among P level, bacteria, and the interaction between P level × bacteria treatments (**Table 2**). Under 50% level treatment, it showed a rise by 88.1% for *B. vallismortis*, 95.2 for *B. tequilensis*, and 130.4 for *B. vallismortis + B. tequilensis* treatment (**Figure 6B**). Under 75% P-level treatment, a similar trend was obtained for *B. vallismortis* and *B. vallismortis + B. tequilensis* (**Figure 6B**). Consequently, total sugars varied greatly among P level, bacteria, and interaction between P level × bacteria treatments (**Table 2**). Under 50% P level treatment, a surge was increased by 85.6% for *B. tequilensis* treatment and a maximum increment by 119.3% for *B. vallismortis + B. tequilensis* treatment. A similar propensity with a 106.6% increment was observed under 75% in combination with *B. vallismortis + B. tequilensis*) treatment (**Figure 6C**). With regard to the accumulated NPK (nitrogen, phosphorus, and potassium) content, the synergistic phosphorus-bacterial interaction greatly enriched the mineral content. The treatment 50% P combined with *B. vallismortis* + *B. tequilensis* intensified the accumulated NPK to 0.065 g, 0.013 g, and 0.050 g DM against 0.021 g, 0.002 g, and 0.013 g DM of the corresponding control treatments, respectively (**Figures 7A–C**).

## 4 Discussion

According to our study, 38 endophytic bacteria were isolated on PVK media from nine medicinal plants. The bacteria can grow on this medium and form a clear zone around the colonies (Amri et al., 2023) due to converting tricalcium phosphate in the medium from insoluble to soluble forms (Zuluaga et al., 2023). The pH of the culture medium used for phosphate solubilization decreased, which may be a sign of organic acids indirectly present. According to some reports, the PSB produces organic acids that cause the culture medium to become more acidic (Benbrik et al., 2020; Sanchez-Gonzalez et al., 2022), which is the primary microbial mechanism by which inorganic P is solubilized (Prabhu et al., 2019).

According to Richardson (2001), the activity of the enzyme phosphatase helps to dissolve the organic phosphates in the soil. Phosphatase activities showed comparable results to those of Bayisa (2023) who recorded alkaline phosphatase activity of *Bacillus Velezensis*. It is significant to note that when inorganic P levels in the growth medium are limited, the innovative production of these enzymes is triggered (Dick et al., 2011). Another advantageous function of several groups of microorganisms is to enable the enzyme nitrogenase to convert molecular nitrogen (N_2_) into ammonia (NH_3_) (Miljaković et al., 2020). The amino acids that are formed from ammonia are then absorbed by plant roots. Radhakrishnan et al. (2017) reported the nitrogenase activities of a number of *Bacillus* species, such as *B. megaterium*, *B. cereus*, *B. pumilus*, *B. circulans*, *B. licheniformis*, *B. subtilis*, *B. brevis*, and *B. firmus*. The nitrogen fixation ability of endophytic *Bacillus* spp. was determined using the ARA, according to Szilagyi-Zecchin et al. (2014). Furthermore, Ikeda et al. (2015) revealed that among the environmental factors, low nitrogen strongly affected the community structures of leaf blade-associated bacteria in rice by increasing the relative abundance of *Bacilli.* Thus, suggesting the potential contribution of their biological nitrogen fixation.

In addition to phosphate solubilization, isolates code 20P and 28P gave a high amount of IAA and were positive for siderophores production, as they exihibited moderate affinity for iron chelation. The IAA phytohormone induces cell elongation, lateral root development, cell division, and differentiation (Goswami et al., 2016). On the other side, siderophore performs a major role in the chelation of micronutrients like iron even in unfavorable conditions (Arora and Verma, 2017). According to previous researches, *Bacillus* spp. have been proven as producers of IAA and siderophores which are crucial for nutrient uptake and plant growth (Walpola and Yoon, 2013; Ribeiro et al., 2018). Additionally, *B. safensis* has been confirmed to produce siderophore and IAA efficiently according to Mukhtar et al. (2017) and Chakraborty et al. (2013), respectively. Producing siderophores, by *Bacillus* spp., helps chelate iron from precipitated forms (FePO_4_) and increases the supply of phosphate ions in the rhizosphere and hence to the plant (Sharma et al., 2013). These characteristics qualified *B. safensis* to promoting plant growth and productivity in both biotic and abiotic environments (Lateef et al., 2015).

In our investigation, the two most potent isolates have been molecularly identified using 16S rRNA as *Bacillus vallismortis* strain 20P and *Bacillus tequilensis* strain 28P with 100% similarity respectively. *Bacillus* spp. are the most prevalent PGPR in nature and are frequently utilized to improve plant growth, development, and yield via secreting bioactive extra-metabolites and nutrients in addition to possessing the capacity to solubilize phosphate in soil.

The present study showed that co-inoculation with PSB; *B. varllismortis* strain 20P and *B. tequilensis* strain 28P generally improved the nutritional canola status, with the consequent significant increase in the development of the plant biomass. Plants co-inoculated with *B. varllismortis* + *B. tequilensis* under 50% P showed significantly higher shoot and root heights, dry masses, leaf area, total dry mass, low rhizosphere pH, higher chlorophyll a, carotenoids, higher soluble sugars, polysaccharides, and total sugars and higher accumulated NPK contents. These results represent a mean of promoting plant growth by increasing the P availability under this P concentration (50% P).

Our findings are consistent with those of Salimpour et al. (2012) who reported enhanced growth, physiological attributes and nutrient uptake of canola as result of applying *Bacillus* sp. and *Thiobacillus* sp. Siddikee et al. (2010) also recorded increased root elongation and dry weight for canola seedlings inoculated with *B. aryabhattai* strain RS341 than un-treated plants.

In support, Park et al. (2015) reported that inoculation of *Kalanchoe daigremontiana* plants with plant growth-promoting rhizobacteria enhanced the plant height, shoot weight, and stem width as well as plant development in terms of increasing number of leaves per plant. Similar consequences were observed in oil palms; the multi-microbial biofertilizer application increased height, chlorophyll, and the number of leaves per plant as compared to the sole chemical fertilizer application (Adiprasetyo et al., 2014). Another valuable explanation of the PSB role is that these bacteria colonize root and rhizosphere soil, which improves microbial and root respiration and root exudation. Finally, this colonization definitely led to enhanced plant height, stem diameter, number of branches, and plant biomass (Khan et al., 2020; Iqbal et al., 2023). Several studies reported that PSB excrete organic acids, which definitely reduces the soil pH that facilitates the P intake (Vazquez et al., 2000; Mehnaz and Lazarovits, 2006; Ali et al., 2017). This fact was a strong proof of our findings which demonstrated the lower pH as the fate of the most effective treatment.

Moreover, Hawkesford et al. (2012) and Youssef et al. (2017) documented that phosphorus enhances the number and mass of roots, the formation of lateral roots, and the root/shoot ratio of plants. As a result, the soil’s ability to absorb nutrients is improved, which in turn promotes plant growth, total chlorophyll, total carbohydrates, and carbon metabolism (Mohamed et al., 2021).

On the contrary, the total dry mass and chl.b showed the maximum level under 75% P combined with *B. varllismortis* +*B. tequilensis*. Accordingly, some studies have documented a reduction in chlorophyll content in combination with PGPR such as (Ajeng et al., 2020). This is most likely due to the non-suitable N needs, as N is a crucial component for chlorophyll content enhancement (Naher et al., 2018). This leads us to hypothesize that the elevated level of Chl.a under 50%P+ *B. varllismortis + B. tequilensis*) simultaneously with the depressed levels of Chl.b under the same treatment, because more N is exhausted in Chl.a formation on the expense of Chl.b.

It has been frequently recorded that enhancing phosphorus supplies has varying effects on carbohydrates content in relation to a source/sink equilibrium in roots and aerial tissues. The accessibility of phosphorus is generally correlated with the metabolism of sugars (Garcia-Caparros et al., 2021). PSB stimulate several metabolic pathways that lead to the synthesis of sucrose, glucose, and fructose. According to results published by Kang et al. (2014), the phosphate-solubilizing *B. megaterium* mj1212 improved photosynthetic pigments and subsequent processes like carbohydrate synthesis, which led to better growth. They also stated that plant-bacterial interactions determine how plants metabolize carbohydrates.

One of the principal roles of PSB is the enhancement of root morphological traits, causing improved nutrient uptake and N fixation (Khan, 2020; de Andrade et al., 2023). In synchronization, Hassan et al. (2017) stated that *Bacillus subtilis* enhanced Pegaga growth by enhancing nutrient intake in the treated plants. An increased N, P, and K contents were observed in black lentils and chickpea plants inoculated with *P. indica* (Nautiyal et al., 2010; Iqbal et al., 2023). PSB converts P from insoluble to soluble available form readily for plant absorption (Reddy, 2014; Gupta et al., 2015). Thus, we can hypothesize that the higher P under co-inoculation of both *Bacillus* strains is most likely due to the optimum P availability specially when being under 50% P treatment.

Generally, these bacillus strains may have the ability to enhance the availability of nutrients such as phosphorus and the production of siderophores and phytohormones (Viruel et al., 2011). In addition, these strains may also be able to colonize the root system and have a positive interaction with the plant, these bacterial characteristics could contribute to growth-promoting effect. The increased nutrient uptake could be ascribed to two main machineries: the first is the synthesis of auxin as IAA, which has a cascading impact on root growth and nutrient intake and, second, to the bacterial propensity to solubilize phosphate, which raises soil P availability. This in turn resulted in enhanced crop yield by promoting plant growth and development (Richardson et al., 2009).

## 5 Conclusion

Overall, co-inoculation of canola with PSB, *Bacillus varllismortis* strain 20P and *Bacillus tequilensis* strain 28P under 50%P showed an upsurge in the yield contributing traits than sole inoculation and the combined inoculation under higher P supply (75 and 1005 P). Our results are a valuable addition to the core of sustainable agricultural practices knowledge and represent one of many steps taken toward the sustainability and maintenance of our planet. It provides information for future field studies of these PSB candidates for development of bioinoculants to improve nutrient availability in soil, reduce chemical fertilizer application, and minimize environmental pollution.

## Data availability statement

The original contributions presented in the study are included in the article/**Supplementary Material**, further inquiries can be directed to the corresponding author/s.

## Author contributions

AN: Data curation, Formal analysis, Investigation, Methodology, Resources, Software, Writing – original draft. ME: Supervision, Validation, Writing – review & editing. OA: Formal analysis, Investigation, Methodology, Software, Writing – original draft. MA: Funding acquisition, Resources, Software, Validation, Formal analysis, Writing – original draft. BA: Funding acquisition, Resources, Software, Validation, Supervision, Writing – original draft. GD: Data curation, Formal Analysis, Investigation, Methodology, Resources, Software, Writing – original draft.
