# Supplementary material for: Endophytic Bacillus vallismortis and Bacillus tequilensis bacteria isolated from medicinal plants enhance phosphorus acquisition and fortify Brassica napus L. vegetative growth and metabolic content
Source: Front Plant Sci. 2024 Mar 22;15:1324538. doi: 10.3389/fpls.2024.1324538 (PMC10995350; doi:10.3389/fpls.2024.1324538)
Supplement: Supplementary file 1 [file DataSheet_1.docx]

**Endophytic *Bacillus vallismortis* and *Bacillus tequilensis* bacteria isolated from medicinal plants enhance phosphorus acquisition and fortify *Brassica napus* L. vegetative growth and metabolic content**

**Aziza Nagah^1^, Mostafa M. El-Sheekh^2*^,** [**Omnia M. Arief**](https://www.cambridge.org/core/search?filters%5BauthorTerms%5D=Omnia%20M.%20Arief&eventCode=SE-AU)**^1^, Mashael Daghash Alqahtani^3^, Basmah M. Alharbi^4^, Ghada E. Dawwam^1^**

^1^Botany and Microbiology Department, Faculty of Science, Benha University, Benha,13518, Egypt.

^2^Botany Department, Faculty of Science, Tanta University, Tanta 31527, Egypt.

^3^Department of Biology, College of Sciences, Princess Nourah bint Abdulrahman University, P.O.BOX 84428, Riyadh 11671, Saudi Arabia.

^4^Basmah M. Alharbi Biology Department, Faculty of Science, University of Tabuk, Tabuk 71491, Saudi Arabia.

***Correspondence:
Mostafa M. El-Sheekh; E mail:** [mostafaelsheikh@science.taanta.edu.eg](mailto:mostafaelsheikh@science.taanta.edu.eg)

**Supplementary Table S1: Thirty-eight endophytic bacteria obtained from roots and leaves of nine medicinal plants gathered from different farms at Qalyubiya Governorate, Egypt**.

| Name of Medicinal plant | Plant part | Number of isolates | Isolate code |
| --- | --- | --- | --- |
| Marjoram*(Origanum majorana)* | Root | 4 | 1P-4P |
|  | Leaf | 2 | 5p,6p |
| Lavender (*Lavandula)* | Root | 3 | 7P-9P |
|  | Leaf | 2 | 10P,11P |
| Mint ([*Mentha spicata*](https://en.wikipedia.org/wiki/Mentha_spicata)*)* | Root | 3 | 12P-14P |
|  | Leaf | 1 | 15P |
| Stevia *(Stevia rebaudiana)* | Root | 5 | 16P-20P |
|  | Leaf | 2 | 21P,22P |
| Rosemary *(Salvia Rosmarinus)* | Root | 3 | 23P-25P |
|  | Leaf | 1 | 26P |
| Kalanchoe *(Kalanchoe blossfeldiana)* | Root | 2 | 27P,28P |
|  | Leaf | 1 | 29P |
| Lemon *(Citrus limon)* | Root | 2 | 30P,31P |
|  | Leaf | 1 | 32P |
| (Nerium) *Nerium oleander* | Root | 2 | 33P,34P |
|  | Leaf | 1 | 35P |
| Roselle *(Hibiscus sabdariffa)* | Root | 2 | 36P,37P |
|  | Leaf | 1 | 38P |

| Treatment | Shoot height (cm) | Shoot FM (g) | Shoot DM (g) | Leaf area (mm^2^ ) | Root height (cm) | Root FM (g) | Root DM (g) | Root Mass ratio | Total DM (g) |
| --- | --- | --- | --- | --- | --- | --- | --- | --- | --- |
| Control | **25.5^a^±4.96** | **12.04^a^±4.05** | **0.88^a^±0.455**  **±** | **98.73^a^± 12.05**  **±** | **6.50^a^±2.24**  **±** | **0.20^a^±0.088**  **±** | **0.04^a^±0.022**  **±** | **0.04^a^±0.020** | **0.92^a^±0.45**  **±** |
| *B. vallismortis* | **27.780^ab^±4.43** | **13.99^ab^±3.13** | **1.17^ab^±0.30**  **±** | **149.30^bcd^±46.84**   \| **46.84** \| \| --- \| \| \| \| \|   **±** | **10.04^abcd^±2.477**  **±** | **0.30^ab^±0.21**  **±** | **0.05^ab^±0.02**  **±** | **0.05^a^±0.03**  **±** | **1.23^ab^±0.29**  **±** |
| *B. tequilensis* | **28.5^ab^±1.50**   \| **28.500** \| \| --- \| \| \| \| \|  \| **28.500** \| \| --- \| \| \| \| \| | **13.15^a^±2.42** | **1.15^ab^±0.21**  **±** | **167.25^bcde^±37.40**   \| **37.40** \| \| --- \| \| \| \| \|   **±** | **10.20^abcd^±2.59**  **±** | **0.63^bcde^±0.24**  **±** | **0.13^cde^±0.05**  **±** | **0.01^bcd^±0.03** | **1.28^ab^±0.23**  **±** |
| (*B.vallismortis+B.tequilensi*s)  *Quilensis* | **30.8^abc^±1.30** | **17.76^bc^±2.32**   \| **2.32** \| \| --- \| \| \| \| \| | **1.49^ab^±0.22**  **±** | **216.34^efg^±45.41**  **±** | **10.20^cd^±1.79** | **0.61^bcde^±0.29**  **±** | **0.15^cde^±0.03**  **±** | **0.09^c^±0.01** | **1.64^bc^±0.25**  **±** |
| 50%P | **27.75^abc^±5.64** | **14.77^abc^±5.31** | **1.25^ab^±0.46**  **±** | **119.21^ab^±23.49**  **±** | **10.56^bcd^±2.38**  **±** | **0.76^abcdef^±0.86** | **0.30^fg^±0.17**  **±** | **0.20^bcdefg^±0.12** | **1.55^abc^±0.47**  **±** |
| \| 50%P+ *B. vallismortis* \| \| --- \| \| 50%P+ *B. tequilensis* \| \| 50%P+(*B.vallismortis+B.tequilensis)* \| | **29.4^abc^±2.04** | **15.58^abc^±3.55** | **1.34^ab^±0.33**  **±** | **165.37^bcde^±72.38**  **±** | **12.76^bc^±4.82**  **±** | **0.63^bcde^±0.23** | **0.10^bcd^±0.04**  **±** | **0.07^abc^±0.03** | **1.44^ab^±0.33**  **±** |
| \| 50%P+ *B. vallismortis* \| \| --- \| \| 50%P+ *B. tequilensis* \| \| 50%P+(*B.vallismortis+B.tequilensis)* \| | **28.7^abc^±2.05** | **14.52^abc^±4.09** | **1.27^ab^±0.31**  **±** | **177.69^cdef^±30.54**  **±** | **10.75^cd^±0.18**  **±** | **0.85^ef^±0.14** | **0.18^def^±0.04**  **±** | **0.13^cde^±0.04** | **1.46^ab^±0.30**  **±** |
| 50%P*+*(*B.vallismortis+B.tequilensis*) | **33.5^c^±4.12** | **23.46^c^±6.94** | **2.12^abc^±0.74** | **296.99^g^±49.86**  **±** | **11.16^bcd^±2.97**  **±** | **0.99^cdef^±0.51** | **0.43^gh^±0.1**  **±** | **0.18^ef^±0.06** | **2.56^c^±0.81**  **±** |
| 75% P | **26.3^ab^±4.47** | **12.48^a^±3.22** | **1.02^ab^±0.33**  **±** | **122.61^abc^±22.79**  **±** | **7.10^ab^±1.88**  **±** | **0.36^abc^±0.24** | **0.39^gh^±0.06**  **±** | **0.29^fg^±0.09** | **1.41^ab^±0.29**   \| **0.33** \| \| --- \| \| \| \| \|   **±** |
| 75%P+ *B. vallismortis* | **27.8^ab^±1.89** | **13.06^a^±2.81** | **1.09^ab^±0.28**  **±** | **143.83^abc^±33.22**  **±** | **11.30^cd^±2.46**  **±** | **0.9 ^cdef^±0.38** | **0.53^h^±0.08**  **±** | **0.33^g^±0.05** | **1.63^bc^±0.32**  **±** |
| 75%P+ *B. tequilensis* | **27.5^a^±3.28** | **13.90^abc^±4.67** | **1.14^ab^±0.41**  **±** | **158.61^bcde^±54.81**  **±** | **14.30^d^±3.05**  **±** | **0.62^bcde^±0.22** | **0.09^bc^±0.04**  **±** | **0.07^abc^±0.03** | **1.22^ab^±0.43**  **±** |
| 75%P+(*B.vallismortis+B.tequilensis*) | **32.32^bc^±2.35** | **19.71^bc^±4.79** | **1.72^ab^±0.47**  **±** | **267.66^fg^±61.60**   \| **61.60** \| \| --- \| \| \| \| \|   **±** | **13.26^d^±2.26**  **±** | **0.54^bcd^±0.17** | **0.12^cde^±0.08**  **±** | **0.07^abc^±0.04** | **1.84^bc^±0.52**  **±** |
| 100 % P | **29.50^abc^±3.43** | **16.19^abc^±4.61** | **1.33^ab^±0.39**  **±** | **173.51^bcdef^±63.33**  **±** | **9.70^abcd^±3.15**  **±** | **0.46^bcd^±0.13** | **0.22^ef^±0.16**  **±** | **0.15^de^±0.04** | **1.55^bc^±0.33**  **±** |
| 100%P+ *B. vallismortis* | **29.1^ab^±3.94** | **16.27^abc^±5.57** | **1.36^ab^±0.49**  **±** | **146.68^abc^±23.35**  **±** | **9.70^bc^±1.52**  **±** | **0.52^bcd^±0.19** | **0.08^bc^±0.03**  **±** | **0.06^ab^±0.02** | **1.44^ab^±0.50**  **±** |
| 100%P+ *B. tequilensis* | **29.8^abc^±2.93** | **16.69^abc^±5.38** | **1.42^ab^±0.50**  **±** | **143.23^bc^±8.45**  **±** | **14.40^cd^±4.32**  **±** | **1.09^f^±0.18** | **0.14^cde^±0.03**  **±** | **0.10^abcde^±0.05** | **1.55 ^abc^±047**  **±** |
| 100%P+(*B.vallismortis+B.tequilensis)*)*)* | **30^abc^±4.06** | **17.42^abc^±5.50** | **1.45^ab^±0.53**  **±** | **211.09^defg^±48.01**  **±** | **9.70^bc^±1.30**  **±** | **0.59^bcde^±0.26** | **0.48^gh^±0.07**  **±** | **0.026^fg^±0.071** | **1.93^bc^±0.53**  **±** |

**Supplementary Table S2:** The Effect of different concentrations of phosphorus (0, 50, 75, and 100%P) alone and/or in combination with *B. vallismortis*, *B. tequilensis* or both bacterial isolates*.* on the vegetative growth of canola ((*Brassica napus L*.) plant


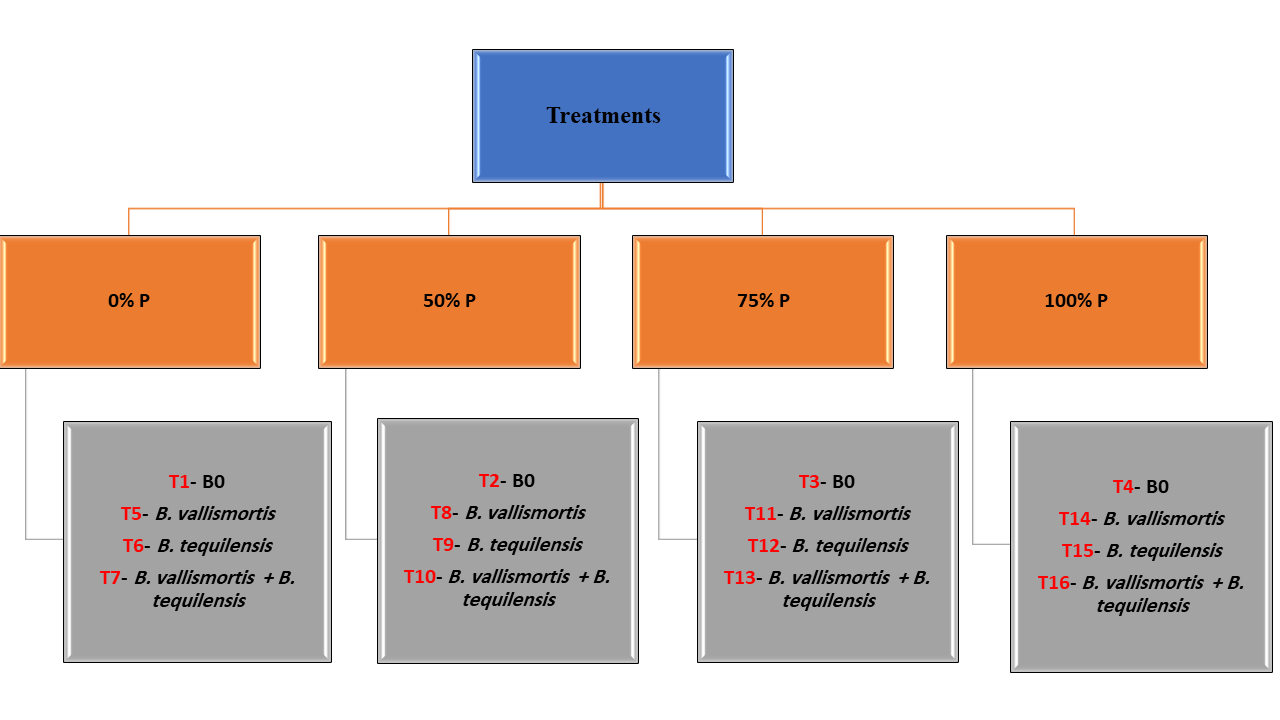


**Supplementary Figure 1**: **Description of the treatments tested for canola (*Brassica napus* L.) growth during the experiment course. *Note: the concentrations of P (as P_2_O_5_), were achieved by the following recipes: 100% P (0.39 g/2 Kg sand: clay soil mixture), 75% P (0.225 g/2 Kg sand: clay soil mixture), 50% P (0.195 g/2 Kg sand: clay soil mixture)**
